# Transcriptomic analysis of the fungal pathogen *Fusarium oxysporum* f. sp. *albedinis* (Foa) during colonization of date palm reveals distinct temporal transcriptional trajectories and uncovers novel candidate effectors

**DOI:** 10.3389/fpls.2026.1954982

**Published:** 2026-10-21

**Authors:** Abdou Lahat Mbaye, Aliou Moussa Diouf, Maimouna Deh, Mohammed Aziz El Houmaizi, Mustapha Barakate, Zineb Rchiad

**Affiliations:** 1 Agrobiosciences Program, College of Agriculture and Environmental Sciences, Mohammed VI Polytechnic University (UM6P), Ben Guerir, Morocco; 2 Laboratory for Agricultural Productions Improvement, Biotechnology and Environment (LAPABE), Faculty of Sciences, University Mohammed First, Oujda, Morocco; 3 Unit of Microbial Biotechnologies, Agrosciences, and Environment (BIOMAGE)-CNRST Labeled Research Unit, Laboratory of Water Sciences, Microbial Biotechnologies, and Natural Resources Sustainability (AQUABIOTECH), Faculty of Sciences-Semlalia, Cadi Ayyad University (UCA), Marrakesh, Morocco; 4 Biosciences Division, UM6P CoreLabs, Mohammed 6 Polytechnic University, Ben Guerir, Morocco

**Keywords:** DEG, effector, *Fusarium oxysporum f.* sp. *albedinis*, necrosis, nlp, RNA-Seq, root pathogen, secreted in xylem

## Abstract

**Background:**

Bayoud disease, caused by *Fusarium oxysporum* f. sp. *albedinis* (Foa), represents a major threat to date palm cultivation and oasis ecosystems. Understanding the molecular mechanisms underlying Foa infection is essential for developing effective control strategies.

**Methods:**

In this study, we performed a temporal transcriptomic analysis of three Foa isolates (FoaER10, FoaER20, and FoaZG10) during colonization of date palm roots at four infection stages (0, 8, 16, and 24 days post-inoculation).

**Results:**

Differential gene expression analysis revealed extensive and stage-dependent transcriptional reprogramming, with the number of significant differentially expressed genes ranging from 4,914 to 6,895 per isolate, and 8,453 unique genes significantly differentially expressed across the three isolates. Comparative analysis identified a core set of 1,079 consistently up-regulated and 123 down-regulated genes, alongside 2,376 genes showing opposite regulation directions between isolates. Functional annotation highlighted carbohydrate-active enzymes (CAZymes), MFS membrane transporters, and candidate effectors including necrosis-inducing-like proteins (NLPs), Hce2/Ecp2, and SIX proteins.

**Discussion:**

This study provides a comprehensive transcriptomic resource for understanding Foa-date palm interactions and establishes a foundation for targeted disease management strategies.

## Introduction

Date palm (*Phoenix dactylifera* L.) is a major crop in arid and semi-arid regions, particularly in North Africa and the Middle East, where it plays a crucial economic and ecological role. However, its production is severely threatened by Bayoud disease, a vascular wilt caused by *Fusarium oxysporum* f. sp. *albedinis* (Foa) (Diouf et al., 2025; Mbaye et al., 2026). This pathogen is responsible for the destruction of millions of palm trees and continues to pose a major risk to oasis ecosystems and agricultural sustainability (Benzohra et al., 2015; Khayi et al., 2023).

The *Fusarium oxysporum* species complex comprises a diverse group of soil-borne fungal pathogens characterized by host-specific formae speciales. These pathogens colonize the vascular system of host plants, leading to systemic infection, tissue necrosis, and eventual plant death (Baayen et al., 2000). In the case of Foa, infection typically begins in the roots and progresses through the xylem, resulting in characteristic symptoms such as leaf wilting, discoloration, and collapse of the crown (Khayi et al., 2023; Rafiqi et al., 2022). Successful infection by *F. oxysporum* relies on a complex interplay of metabolic adaptation, secretion of cell wall-degrading enzymes (CWDEs), production of toxins, and deployment of effector proteins. These effectors, including Secreted In Xylem (SIX) proteins and necrosis-inducing proteins (NLPs), play key roles in host colonization and immune modulation (De Wit et al., 2009; D. A. Jones et al., 2018; Rep et al., 2004). In well-studied pathosystems such as *F. oxysporum* f. sp. *lycopersici*, specific effectors have been shown to interact with host resistance genes, illustrating the importance of effector-mediated host–pathogen interactions (Jenkins et al., 2021; J. D. G. Jones and Dangl, 2006).

Despite its importance, the molecular mechanisms underlying Foa pathogenicity in date palm remain poorly understood. Recent genomic studies have provided insights into the genetic architecture of Foa, including the identification of genes potentially associated with pathogenicity (Ayada et al., 2022; Khayi et al., 2023). However, genomic data alone do not capture the dynamic regulation of gene expression during infection. Transcriptomic approaches, particularly RNA sequencing (RNAseq), have become powerful tools for studying host–pathogen interactions by enabling the identification of differentially expressed genes (DEGs) during infection. In *Fusarium* species, RNA-seq has been successfully used to characterize infection-related gene expression, revealing the activation of pathways involved in metabolism, cell wall degradation, and effector secretion (Huang et al., 2019; Thatcher et al., 2016; Yang et al., 2022). However, to date, no comprehensive temporal transcriptomic analysis of Foa during date palm infection has been reported, particularly in a comparative framework involving multiple isolates. Furthermore, the timing and dynamics of gene expression associated with pathogenicity remain largely unexplored in this pathosystem.

In this study, we performed a comparative temporal transcriptomic analysis of three Foa isolates during colonization of date palm roots at four infection stages (0, 8, 16, and 24 days post-inoculation). By integrating differential gene expression analysis, functional annotation, and RTqPCR validation, we aimed to (1) characterize stage-dependent transcriptional changes during infection, (2) identify candidate genes associated with pathogenicity, and (3) compare transcriptional dynamics among isolates. This work provides new insights into the molecular basis of Foa date palm interactions and establishes a foundation for future functional studies aimed at understanding pathogenicity mechanisms and improving disease management strategies.

## Materials and methods

### Isolate sources and growth conditions

Three *Fusarium oxysporum* f. sp. *albedinis* (Foa) isolates (FoaER10, FoaER20, and FoaZG10) originating from diseased date palm roots in Morocco were used in this study. Isolates were molecularly authenticated using the species- and *forma specialis*-specific PCR primer pair FOA28 (5′- ATCCCCGTAAAGCCCTGAAGC-3’) and TL3 (5′- GGTCGTCCGCAGAGTATACCGGC-3’) which amplifies a diagnostic 400 bp genomic region unique to the Foa lineage (Ferniah et al., 2014; Tantaoui et al., 1996). Amplicons were sequenced, exhibiting >99.5% sequence identity to Foa reference sequences in NCBI GenBank (Mbaye et al., 2026). The sequences were deposited in the NCBI database under BioSample accession numbers SAMN49516559 (FoaER10), SAMN49517112 (FoaER20), and SAMN49517222 (FoaZG10). Forma specialis-level identity was further confirmed via pathogenicity bioassays on susceptible date palm seedlings (Phoenix dactylifera). All three isolates induced characteristic vascular wilt symptoms, confirming pathogenicity. Disease Severity Index (DSI) and incubation metrics demonstrated distinct virulence hierarchies, identifying FoaER10 as the most aggressive strain, followed by FoaER20 and FoaZG10 (**Table 1** in **Supplementary File 1**).

**Table 1:** Primer sequences for the targeted genes.

Primers name	Sequence	Target
SIX1_F	ATGGCGCCCTATAGCATGG	Secreted in Xylem1 (SIX1) gene, partial cds
SIX1_R	TTGGAGTTCCCTAGACGCCC	
SIX9_F	GAAGCTTCTAGCAGGCGCAG	Secreted in Xylem9 (SIX9) gene, complete cds
SIX9_R	TTTACAAGCTCCCGTGTGCC	
H9L3915275_F (NLP)	CTCTCCCATCACCGAGTCTT	Necrosis Inducing protein domain-containing Protein (FOBCDRAFT_269836), partial mRNA
H9L3915275_R (NLP)	AGTATGGTAGATACCCCATCGCA	
H9L3904485_F (NLP)	TCTGGCATCCTTGCATCACC	Necrosis Inducing protein (FOBCDRAFT_282248), partial mRNA
H9L3904485_R (NLP)	AGAAACTGCCATGCCAACAAC	

### Colonization of date palm roots by Foa

Spore inoculation assays were performed to evaluate the colonization of date palm roots by three Foa isolates (FoaER10, FoaER20, and FoaZG10), following a modified protocol based on Williams et al. (2016). Three-month-old date palm seedlings of cultivar Boufgouss, derived from open-pollinated seeds, were used for inoculation assays; host genetic heterogeneity arising from this open-pollinated background is addressed statistically through biological replication rather than through tissue pooling. The experiment followed a completely randomized design. For each of the three Foa isolates, a single conidial suspension (1 × 10^6^ spores mL^-^¹, prepared from a 7-day-old PDA culture) was prepared. All seedlings destined for that isolate, twelve in total, spanning all four post-inoculation sampling points and their three biological replicates, were immersed together in this single suspension and incubated for 6 hours at 28 °C in the dark. Following this inoculation period, seedlings were removed from the suspension, transferred to pots containing sterile peat substrate, and maintained under controlled growth conditions (28 °C). Three seedlings (biological replicates) were destructively sampled immediately upon removal from the spore suspension, constituting the 0 dpi (6 hours post-inoculation) time point, while the remaining seedlings were maintained and destructively sampled at 8, 16, and 24 days post-inoculation (three biological replicates per time point). This yielded 12 seedlings per isolate and 36 RNA-seq libraries in total across the three isolates. Because all seedlings for a given isolate were inoculated from the same suspension, sample-to-sample variation in inoculum quality is not confounded with time point within an isolate. A complete sample-metadata table listing sample ID, isolate, time point, and biological replicate is provided in **Supplementary File 2**. Plants were monitored over a period of 4 weeks. Disease progression was assessed every 7 days using a visual disease severity scale based on symptom development, including chlorosis, wilting, necrosis, and plant death.

### RNA isolation, library construction, Illumina sequencing, and read processing

Root tissue samples were harvested at four distinct colonization stages: 0 dpi (defined specifically as 6 hours post-inoculation, 6 hpi, capturing initial fungal attachment), 8 dpi (early colonization), 16 dpi (mid-stage tissue invasion), and 24 dpi (late-stage colonization). For each condition, tissues were pooled to generate three independent biological replicates. Total RNA was extracted from each replicate using the RNeasy Mini Kit (Qiagen) according to the manufacturer’s instructions. RNA concentration and purity were assessed using a NanoDrop spectrophotometer (Thermo Fisher Scientific), and RNA integrity was evaluated using agarose gel electrophoresis and/or a Bioanalyzer system. For each sample, 3 µg of high-quality RNA was used for cDNA library construction. Sequencing libraries were prepared using a standard Illumina-compatible protocol, including rRNA depletion, fragmentation, first- and second-strand cDNA synthesis, end repair, adaptor ligation, and PCR amplification. Libraries were size-selected and validated prior to sequencing on an Illumina HiSeq platform. Raw sequencing reads were subjected to quality control using FastQC (v0.11.9). Adapter sequences, reads containing poly-N regions, and low-quality reads were removed using fastp (v0.23.2) to obtain high-quality clean reads. Quality metrics, including Q30 and GC content, were calculated to assess sequencing performance. Only high-quality reads were retained for downstream analyses. The RNA sequencing data generated in this study have been deposited in the NCBI Sequence Read Archive (SRA) under the BioProject accession numbers PRJNA1422174 (FoaER10), PRJNA1491631 (FoaER20), and PRJNA1422436 (FoaZG10), corresponding to the three Foa isolates. The dataset also includes a negative control. Additional accession numbers will be provided upon completion of data submission.

### Differential gene expression analysis and annotation

Clean reads were aligned to the chromosome-scale genome assembly of *Fusarium oxysporum* f. sp. *albedinis* strain Foa44 (Khayi et al., 2023; BioProject PRJNA658960, BioSample SAMN15893572, GenBank/DDBJ/ENA accession JACSDM000000000) using HISAT2 (v2.2.1) with default parameters. Gene-level read counts for the 36 libraries were obtained using HTSeq and compiled into a count matrix for downstream analysis. For each Foa isolate, differential expression was assessed independently using DESeq2 (v1.30.0) with the design ~ time, a four-level factor corresponding to the four post-inoculation sampling points (0, 8, 16, and 24 dpi), with 0 dpi (6 hpi) as the reference level. Three conditions were tested per isolate, each comparing infected root samples at a later time point against infected root samples at 0 dpi from the same isolate: 8 dpi vs. 0 dpi, 16 dpi vs. 0 dpi, and 24 dpi vs. 0 dpi.Raw read counts were normalized using the DESeq2 internal normalization method based on size factors to account for differences in sequencing depth (Love et al., 2014). Genes with a raw p-value < 0.05 and an absolute log_2_ fold change (|log_2_FC|) ≥ 1 were considered significantly differentially expressed (DE). Functional enrichment analysis for pathogenicity-associated genes among up- and down-regulated DEGs was performed to identify overrepresented biological processes. Gene Ontology (GO) enrichment analysis was conducted using GOseq (v2.12) (Young et al., 2010), which accounts for gene length bias in RNA-seq data. Enriched GO terms with p-values < 0.05 were considered statistically significant.

### Reverse transcription quantitative PCR (RT-qPCR) verification

Quantitative real-time PCR (RT-qPCR) was performed to validate the RNA-seq expression profiles of four selected candidate pathogenicity genes: SIX1 (H9L39_19845), SIX9 (H9L39_17240), and two necrosis-inducing-like protein genes, NLP (H9L39_15275) and NLP (H9L39_04485). cDNA was synthesized from total RNA extracted from biological replicates of the three Foa isolates (FoaER10, FoaER20, and FoaZG10) at 16 dpi, the same infected timepoint used for RNA-seq. Amplification reactions were carried out in technical triplicates on a CFX96 Real-Time System, Bio-Rad using SYBR Green PCR Master Mix.

To adhere to MIQE guidelines, primer efficiencies for each target and reference gene were evaluated using a five-point serial dilution standard curve, ensuring amplification efficiencies between 90% and 110% ( $R^{2}>0.98$). The internal reference gene *ACTIN* was selected based on its proven expression stability across all experimental conditions. No-template controls (NTC) and minus-reverse-transcriptase (-RT) controls were included in every run to rule out reagent contamination and genomic DNA amplification. Relative gene expression changes were calculated using the $2^{-\text{ΔΔ}C_{T}}$ method (Livak and Schmittgen, 2001), with the 0 dpi (6 hours post-inoculation) infected samples of each isolate serving as the baseline calibrator condition, consistent with the RNA-seq differential expression analysis.

## Results

### RNA sequencing and data quality assessment

A total of 36 transcriptome libraries were generated from three Foa isolates (FoaER10, FoaER20, and FoaZG10), including 36 infected date palm root samples collected across four time points (0, 8, 16, and 24 days post-inoculation), with three biological replicates per isolate timepoint combination. High-throughput sequencing produced approximately 190.6 Gb of raw data. Quality assessment indicated high sequencing reliability, with Q30 values exceeding 89% across all samples (mean ± SD: 94.9% ± 1.8%) and consistently low error rates (0.01–0.02%). GC content ranged from 43.2% to 55.1% (mean: 51.3% ± 3.2%), consistent with the expected composition of the *Fusarium oxysporum* genome (**Table 2**, **Figure 1**; **Supplementary File 1**).

**Table 2:** Summary statistics of sequencing quality across all RNA-seq libraries.

Parameter	Range	Mean ± SD	Median
Raw reads (million)	4.2 – 90.1	~34.5 ± 18.2	39.0
Clean reads (%)	89.8 – 97.9	~95.2 ± 2.3	95.7
Error rate (%)	0.01 – 0.02	~0.01 ± 0.002	0.01
Q30 (%)	89.8 – 96.7	~94.9 ± 1.8	95.4
GC content (%)	43.2 – 55.1	~51.3 ± 3.2	52.1

**Figure 1:**
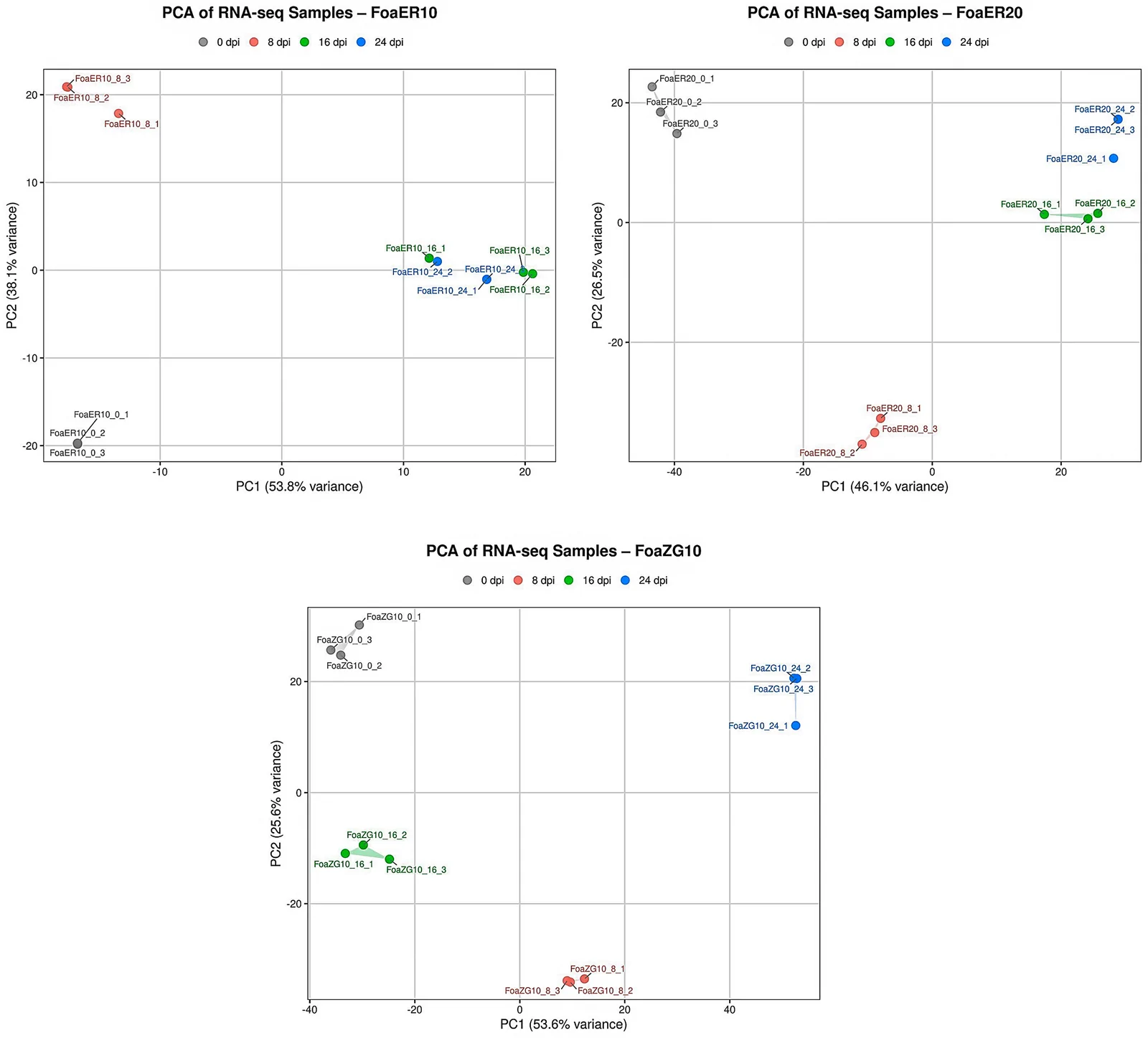
Principal Component Analysis (PCA) of RNA-seq samples across infection stages in Foa isolates. PCA score plots display global transcriptomic variance and sample clustering across four time points (0, 8, 16, and 24 dpi) for FoaER10, FoaER20, and FoaZG10. Each point represents an individual biological replicate (n = 3 per time point), color-coded by infection stage: 0 dpi (grey), 8 dpi (red), 16 dpi (green), and 24 dpi (blue). Axes represent Principal Component 1 (PC1) and Principal Component 2 (PC2).

### Stage-dependent DEG profiles across Foa isolates during host colonization

To characterize the dynamics of molecular responses during host tissue colonization, differential gene expression was analyzed independently at each infection stage (8, 16, and 24 dpi) relative to the 0 dpi baseline, for the three Foa isolates (FoaER10, FoaER20, and FoaZG10). Across all isolates, a total of 8,453 unique genes were significantly differentially expressed in at least one isolate at one or more timepoints, with the number of significant DEGs per isolate ranging from 4,914 (FoaER20) to 6,895 (FoaZG10). Integrated visualization using heatmaps (**Figure 2**) and volcano plots (**Figure 3**) demonstrated highly divergent, isolate-specific, and stage-dependent transcriptional trajectories, with pathogenicity-associated genes (SIX/NLP/Hce2 effectors, priority CAZymes, and other priority Pfam domains) consistently among the most strongly up- and down-regulated genes at every stage.

**Figure 2:**
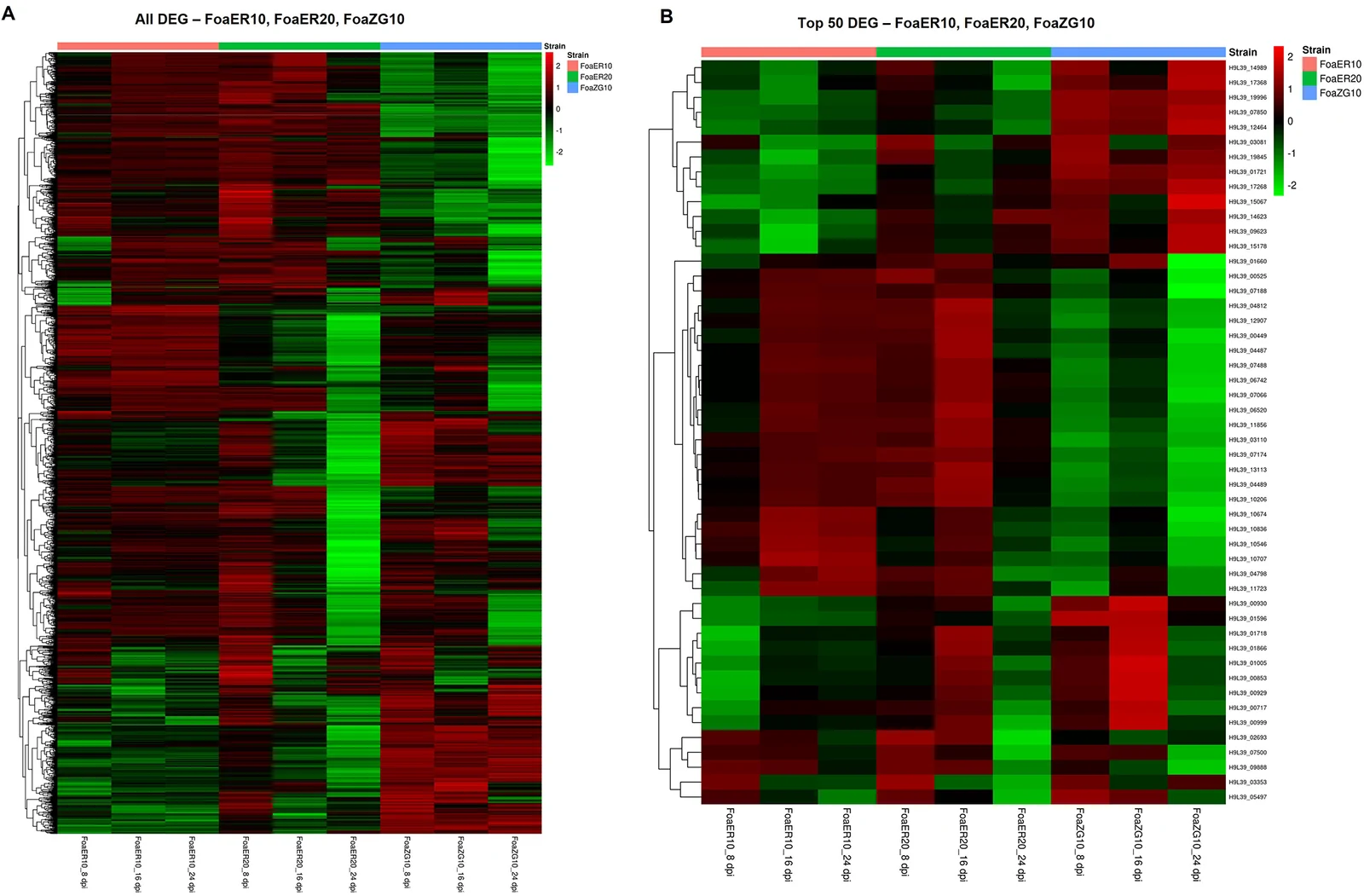
Hierarchical clustering heatmaps of DEGs across infection time points in three Foa isolates. **(A)** Global DEG Expression Heatmap: Expression profiles of all DEGs across FoaER10, FoaER20, and FoaZG10 at 8, 16, and 24 days post-inoculation (dpi). **(B)** Top 50 DEGs expression heatmap: Expression profiles and hierarchical clustering of the top 50 most significantly differentially expressed genes. Columns represent infection time points grouped by isolate, highlighted by the top color bar: FoaER10 (red), FoaER20 (green), and FoaZG10 (blue). Relative expression levels are represented on a Z-score scale ranging from strong upregulation (red, +2) to strong downregulation (green, -2).

**Figure 3:**
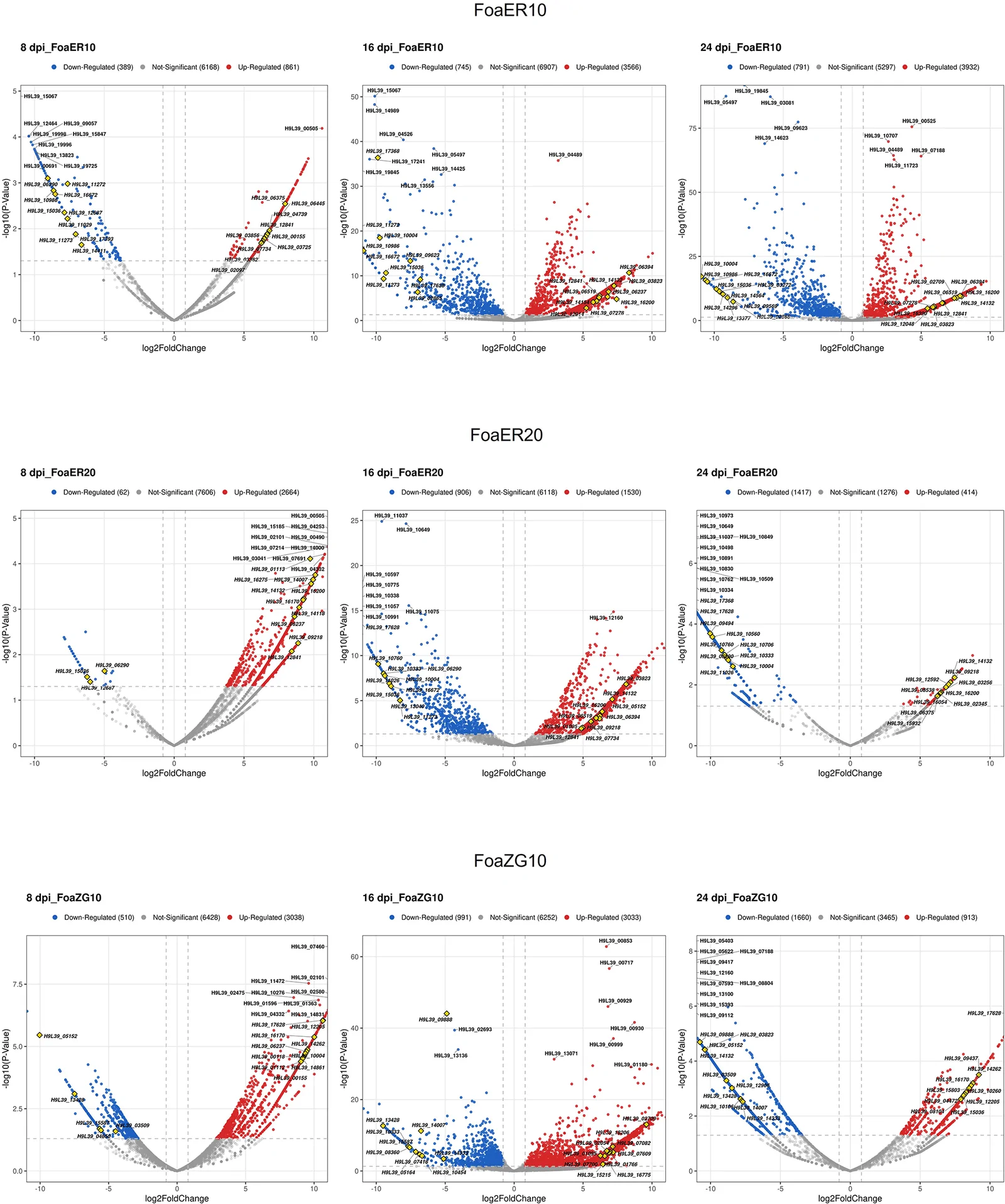
Volcano plots illustrating stage-specific differential gene expression profiles across Foa isolates. Panels display transcriptomic changes at 8 dpi (left column), 16 dpi (middle column), and 24 dpi (right column) for isolates FoaER10 (top row), FoaER20 (middle row), and FoaZG10 (bottom row), each measured against the 0 dpi baseline within the same isolate. Vertical and horizontal dashed lines demarcate the significance thresholds used throughout this study (|log_2_FC| ≥ 1, P ≤ 0.05). Red points represent significantly up-regulated DEGs, blue points represent significantly down-regulated DEGs, and grey points indicate genes not meeting the significance thresholds. The number of genes in each category is given in the legend above each panel. Gold diamond markers indicate candidate pathogenicity-associated genes, SIX, NLP, and Hce2/Ecp2 effector homologs, priority CAZymes, and other priority Pfam domains (MFS transporters, Lipase_3, Abhydrolase), among the top 10 most significantly up-regulated and top 10 most significantly down-regulated genes in each panel, labeled with their corresponding H9L39 locus tag.

The full-DEG heatmap (**Figure 2A**) shows that, although each isolate’s 8/16/24 dpi columns cluster together internally, expression trajectories diverge substantially between isolates: numerous gene blocks that are predominantly red (up-regulated) across the FoaER10 or FoaER20 columns appear predominantly green (down-regulated) across the FoaZG10 columns, and vice versa, particularly at 24 dpi. This pattern is consistent with the Venn diagram analysis, which identified 2,376 genes up-regulated in at least one isolate but down-regulated in another, indicating that a substantial fraction of the shared transcriptional response is regulated in opposite directions depending on the isolate, rather than differing only in magnitude or timing. Among the top 50 most significant DEGs overall (**Figure 2B**), several genes showed isolate-specific reversal of expression direction across the time course, for example, H9L39_00930, H9L39_01718, and H9L39_00717 shift from strongly down-regulated (green) in FoaER10 to strongly up-regulated (red) in FoaZG10, while others, such as H9L39_19845 (SIX1), were consistently up-regulated across all three isolates, reaching some of the highest fold-changes in the dataset by 24 dpi.

At 8 dpi (early colonization), the three isolates already showed substantially different transcriptional magnitudes. FoaER20 displayed the most extensive early response, with 2,726 total DEGs (62 down-regulated, 2,664 up-regulated), followed by FoaZG10 with 3,549 total DEGs (510 down-regulated, 3,039 up-regulated). FoaER10 showed a more modest early response, with 1,250 total DEGs (389 down-regulated, 861 up-regulated). Across all three isolates at this stage, up-regulation dominated the response, and several pathogenicity-associated genes were already among the most significantly induced transcripts (H9L39_06375, H9L39_06445 in FoaER10; H9L39_04332, H9L39_09218 in FoaER20; H9L39_06237, H9L39_10004 in FoaZG10).

The 16 dpi timepoint (mid-stage tissue invasion) marked a substantial expansion of the transcriptional response in FoaER10 and FoaZG10. FoaER10 reached 4,313 total DEGs (745 down-regulated, 3,568 up-regulated), and FoaZG10 reached 4,028 total DEGs (992 down-regulated, 3,036 up-regulated), its largest response of the three timepoints. FoaER20, in contrast, showed a marked contraction relative to its 8 dpi peak, with 2,438 total DEGs (904 down-regulated, 1,534 up-regulated), indicating an early-onset, early-peaking transcriptional strategy rather than a mid-stage maximum. This mid-stage divergence is visible in the heatmap as a shift in the color balance of the FoaER20 column block relative to FoaER10 and FoaZG10 at the corresponding timepoint.

At 24 dpi (late-stage colonization), the isolates diverged further. FoaER10 showed its largest and most up-regulation-dominated response, with 4,723 total DEGs (791 down-regulated, 3,932 up-regulated). FoaZG10 showed a contraction relative to 16 dpi but remained substantially active, with 2,575 total DEGs (1,660 down-regulated, 915 up-regulated). FoaER20 showed the most pronounced late-stage shift toward repression, with 1,832 total DEGs (1,416 down-regulated, 416 up-regulated), reversing its earlier up-regulation-dominated profile, visible in the heatmap as the FoaER20_24dpi column trending markedly greener relative to its own earlier timepoints.

Comparative analysis across isolates and stages, supported by Venn diagram overlap analysis, identified a core set of 1,079 genes consistently up-regulated and 123 genes consistently down-regulated across all three isolates when significant DEGs were pooled across the full time course, alongside substantial isolate-specific and even direction-reversed responses as described above. Taken together, these dynamics reveal three distinct temporal transcriptional strategies: FoaER20 shows an early, up-regulation-dominated onset that peaks at 8 dpi before contracting and shifting toward repression by 24 dpi. FoaER10 shows a progressive, sustained expansion of its transcriptional response from 8 to 24 dpi, culminating in its largest and most up-regulation-dominated response at the latest stage; and FoaZG10 shows sustained, broadly up-regulation-dominated activity through 16 dpi before a partial late-stage contraction. These isolate-specific temporal profiles, together with the substantial subset of genes showing isolate-dependent reversal in regulation direction, indicate fundamental differences in both the timing and regulatory logic of host colonization among date palm Foa isolates.

### Pathogenicity-associated genes and functional annotation of DEGs in Foa

Functional annotation of DEGs identified several gene groups potentially associated with pathogenicity across the three Foa isolates during the infection time course (**Table 3**; **Supplementary File 3**). Consistent with the genome-wide GO enrichment analysis restricted to pathogenicity-associated genes, the DEG set was significantly enriched for carbohydrate-active enzyme (CAZyme) activity, including chitinase activity, chitin catabolic process, cellulose binding, and polysaccharide catabolic process, as well as transmembrane transport and oxidoreductase activity, indicating that cell wall degradation, nutrient/metabolite transport, and redox-related detoxification are among the principal molecular strategies deployed during host colonization (B. C. King et al., 2011).

**Table 3:** Differentially expressed selected based on known or putative roles in pathogenicity, representing distinct functional categories (effectors, toxins, cell wall-degrading enzymes, transporters, and detoxification genes) across the three Foa isolates.

Strain	ID	Protein_id	product	Secreted	Pfam_pathogenicity_flag
FoaER10	H9L39_03256	KAK2485276.1	hypothetical protein	yes	Hce2_Ecp2_effector
FoaER10	H9L39_04485	KAK2482693.1	hypothetical protein	yes	NLP_necrosis_toxin
FoaER10	H9L39_13576	KAK2473616.1	hypothetical protein	yes	NLP_necrosis_toxin
FoaER10	H9L39_15275	KAK2473100.1	hypothetical protein	yes	NLP_necrosis_toxin
FoaER10	H9L39_17240	KAK2471009.1	hypothetical protein	yes	SIX9_Secreted_In_Xylem9
FoaER10	H9L39_19845	KAK2468199.1	hypothetical protein	yes	SIX1_Secreted_In_Xylem1
FoaER10	H9L39_04351	KAK2482559.1	hypothetical protein	yes	Lipase_3
FoaER10	H9L39_09391	KAK2480017.1	putative lipase atg15	yes	Lipase_3
FoaER10	H9L39_04074	KAK2482282.1	hypothetical protein	yes	MFS_transporter
FoaER10	H9L39_08177	KAK2478803.1	hypothetical protein	yes	MFS_transporter
FoaER10	H9L39_01113	KAK2487186.1	hypothetical protein	yes	priority_CAZyme
FoaER10	H9L39_01963	KAK2488036.1	hypothetical protein	yes	other_CAZyme
FoaER10	H9L39_02451	KAK2484471.1	hypothetical protein	yes	other_CAZyme
FoaER10	H9L39_02457	KAK2484477.1	hypothetical protein	yes	other_CAZyme
FoaER10	H9L39_02608	KAK2484628.1	hypothetical protein	yes	Abhydrolase_1
FoaER10	H9L39_02637	KAK2484657.1	hypothetical protein	yes	Abhydrolase_6
FoaER20	H9L39_03256	KAK2485276.1	hypothetical protein	yes	Hce2_Ecp2_effector
FoaER20	H9L39_04485	KAK2482693.1	hypothetical protein	yes	NLP_necrosis_toxin
FoaER20	H9L39_15275	KAK2473100.1	hypothetical protein	yes	NLP_necrosis_toxin
FoaER20	H9L39_17240	KAK2471009.1	hypothetical protein	yes	SIX9_Secreted_In_Xylem9
FoaER20	H9L39_19845	KAK2468199.1	hypothetical protein	yes	SIX1_Secreted_In_Xylem1
FoaER20	H9L39_04351	KAK2482559.1	hypothetical protein	yes	Lipase_3
FoaER20	H9L39_09391	KAK2480017.1	putative lipase atg15	yes	Lipase_3
FoaER20	H9L39_18292	KAK2470144.1	hypothetical protein	yes	MFS_transporter
FoaER20	H9L39_00231	KAK2486304.1	hypothetical protein	yes	other_CAZyme
FoaER20	H9L39_00898	KAK2486971.1	hypothetical protein	yes	priority_CAZyme
FoaER20	H9L39_01113	KAK2487186.1	hypothetical protein	yes	priority_CAZyme
FoaER20	H9L39_01963	KAK2488036.1	hypothetical protein	yes	other_CAZyme
FoaER20	H9L39_05841	KAK2484049.1	hypothetical protein	yes	Abhydrolase_1
FoaER20	H9L39_07700	KAK2482061.1	hypothetical protein	yes	Abhydrolase_6
FoaZG10	H9L39_03256	KAK2485276.1	hypothetical protein	yes	Hce2_Ecp2_effector
FoaZG10	H9L39_04485	KAK2482693.1	hypothetical protein	yes	NLP_necrosis_toxin
FoaZG10	H9L39_05975	KAK2484183.1	hypothetical protein	yes	NLP_necrosis_toxin
FoaZG10	H9L39_15275	KAK2473100.1	hypothetical protein	yes	NLP_necrosis_toxin
FoaZG10	H9L39_17240	KAK2471009.1	hypothetical protein	yes	SIX9_Secreted_In_Xylem9
FoaZG10	H9L39_19845	KAK2468199.1	hypothetical protein	yes	SIX1_Secreted_In_Xylem1
FoaZG10	H9L39_04351	KAK2482559.1	hypothetical protein	yes	Lipase_3
FoaZG10	H9L39_09391	KAK2480017.1	putative lipase atg15	yes	Lipase_3
FoaZG10	H9L39_00309	KAK2486382.1	hypothetical protein	yes	other_CAZyme
FoaZG10	H9L39_00359	KAK2486432.1	hypothetical protein	yes	other_CAZyme
FoaZG10	H9L39_00898	KAK2486971.1	hypothetical protein	yes	priority_CAZyme
FoaZG10	H9L39_01113	KAK2487186.1	hypothetical protein	yes	priority_CAZyme
FoaZG10	H9L39_05841	KAK2484049.1	hypothetical protein	yes	Abhydrolase_1
FoaZG10	H9L39_07700	KAK2482061.1	hypothetical protein	yes	Abhydrolase_6

This transport-dominated signature is directly reflected in the pathogenicity genes highlighted individually in the volcano plot analysis (**Figure 3**): of the sixteen candidate pathogenicity genes annotated across the nine isolate-timepoint panels, ten are major facilitator superfamily (MFS) transporters (H9L39_06375, H9L39_06445, H9L39_09218, H9L39_06237, H9L39_14132, H9L39_07734, H9L39_12841, H9L39_07082, H9L39_07609, and H9L39_14262) (Del Sorbo et al., 2000), consistent with MFS transporters being the single largest category of pathogenicity-associated genes identified in this dataset overall. Two additional genes (H9L39_06519 and H9L39_07278) carry abhydrolase_1/abhydrolase_6 domains, associated with ester and lipid hydrolysis, and one (H9L39_10004) is a priority CAZyme of family GH7 (a cellulase family), mirroring the CAZyme-mediated cell wall degradation signal identified genome-wide. Several priority CAZyme-encoding genes were differentially expressed across isolates, most notably H9L39_01113 (also GH7), which was significantly up-regulated at 8 dpi in all three isolates (FoaER10: log2FC = 4.28; FoaER20: log2FC = 9.74; FoaZG10: log2FC = 8.10), representing one of the earliest and most consistent transcriptional responses across the dataset. H9L39_00898 (family GH18, a chitinase family) was also strongly induced at 8 dpi, reaching significance in FoaZG10 (log2FC = 6.23). Genes annotated with Lipase_3 (H9L39_04351) and abhydrolase_6 (H9L39_02637) domains were also significantly up-regulated in a strain-dependent manner (e.g., H9L39_04351: log2FC = 5.63 in FoaER20 and 7.04 in FoaZG10 at 8 dpi).

Importantly, several genes encoding known Fusarium effector and toxin protein families were differentially expressed across isolates, including four necrosis-inducing-like proteins (NLPs: H9L39_04485, H9L39_13576, H9L39_15275, and H9L39_05975) (Oome and Van Den Ackerveken, 2014), a Hce2/Ecp2-family effector (H9L39_03256) (Stergiopoulos et al., 2012), and two Secreted In Xylem proteins, SIX1 (H9L39_19845) and SIX9 (H9L39_17240), well-characterized virulence-associated effector families in *Fusarium oxysporum* (Houterman et al., 2007; Rocafort et al., 2020). These genes showed markedly isolate- and stage-dependent expression, including cases of direction reversal across timepoints within the same isolate. SIX1 (H9L39_19845), for example, was significantly down-regulated in FoaER10 at 8 dpi (log2FC = −6.53) and 24 dpi (log2FC = −7.82) but significantly up-regulated at 16 dpi (log2FC = 3.01), whereas in FoaZG10 it was significantly up-regulated at all three timepoints (log2FC = 3.72, 8.49, and 2.75 at 8, 16, and 24 dpi, respectively). SIX9 (H9L39_17240) showed a comparable pattern, being significantly down-regulated at all three timepoints in FoaER10 but significantly up-regulated at 16 and 24 dpi in FoaER20 and FoaZG10. This isolate-specific reversal in effector gene regulation is consistent with the broader pattern identified through Venn diagram analysis, in which 2,375 genes were found to be up-regulated in at least one isolate and down-regulated in another, and further supports the interpretation that Foa isolates deploy distinct, and in some cases opposing, regulatory strategies around a shared core set of candidate pathogenicity genes.

Overall, the functional annotation of DEGs highlights CAZyme-mediated cell wall degradation, membrane transport, oxidoreductase/detoxification activity, and effector gene deployment (NLPs, SIX1, SIX9, and Hce2/Ecp2) as the principal pathogenicity-associated processes differentially regulated during Foa infection, with the MFS transporter family emerging as a particularly prominent and recurring feature across both the genome-wide enrichment analysis and the individual genes highlighted at the volcano-plot level, and with expression timing and direction varying substantially between isolates rather than following a uniform program.

### Overlapping DEGs in *in planta* datasets of the three strains link to pathogenicity

To capture the transcriptional dynamics of Foa pathogenesis, we performed a comparative analysis of in planta transcriptomes across three infection stages (8, 16, and 24 dpi) relative to the 0 dpi baseline for each isolate. Host colonization triggered massive transcriptional remodeling, yielding a total of 8,453 unique differentially expressed genes (DEGs) across the three isolates, with per-isolate totals ranging from 4,914 (FoaER20) to 6,895 (FoaZG10) (**Figure 4**). A three-way comparison of up-regulated DEGs across isolates identified a conserved core of 1,079 genes (14.4%) shared by all three isolates, likely driving fundamental pathogenicity functions, while the remaining up-regulated DEGs were substantially isolate-specific (911 genes, 12.1%, unique to FoaER10, 242 genes, 3.2%, unique to FoaER20, and 2,080 genes, 27.7%, unique to FoaZG10) (**Figure 4**). Down-regulated DEGs showed a smaller but still substantial conserved core of 123 genes (3.7%) shared among all three isolates, with isolate-specific down-regulated genes numbering 164 (4.9%) in FoaER10, 547 (16.5%) in FoaER20, and 1,614 (48.6%) in FoaZG10., .

**Figure 4:**
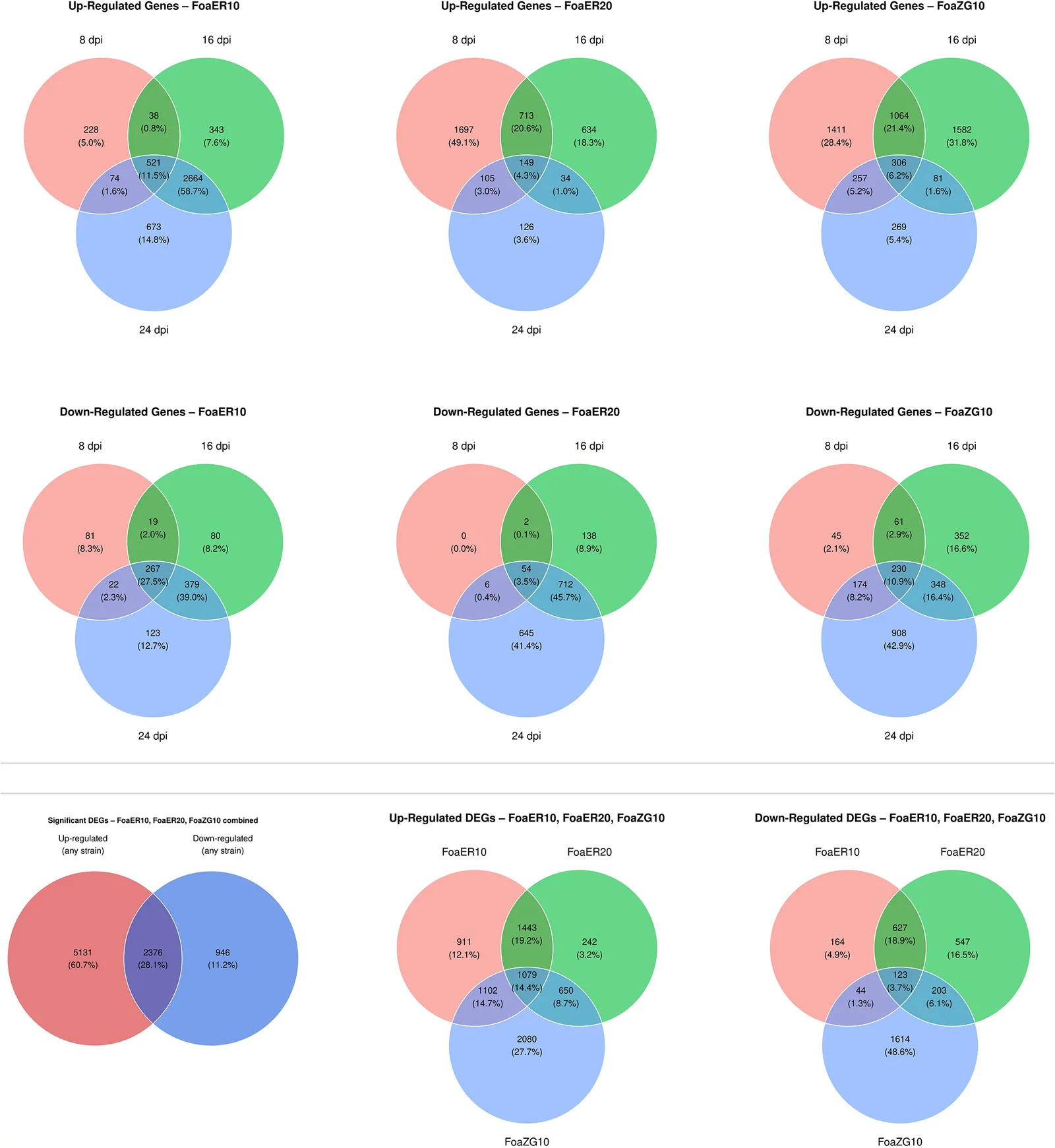
Venn diagrams illustrating the temporal dynamics and cross-strain overlaps of differentially expressed genes (DEGs) in Foa isolates. Top and middle panels display the distribution of unique and shared up-regulated (top row) and down-regulated (middle row) DEGs across three infection time points (8, 16, and 24 dpi) for each strain individually (FoaER10, FoaER20, and FoaZG10). Bottom panels illustrate cross-strain comparisons: the overall overlap between total up-regulated and down-regulated DEGs combined across all strains (bottom left), alongside three-way comparisons showing strain-specific and core shared up-regulated (bottom middle) and down-regulated (bottom right) DEGs across FoaER10, FoaER20, and FoaZG10. Values indicate DEG counts with percentage proportions shown in parentheses.

Temporal profiling within each isolate (comparing which genes were differentially expressed at 8, 16, and 24 dpi) revealed that the three isolates employ markedly different kinetic strategies, though all three showed substantial gene sharing between at least some timepoints, contrary to a strictly non-overlapping model.

FoaER10 showed a late-dominant, cumulative up-regulation pattern: the largest single contribution came from genes shared between 16 and 24 dpi specifically (2,664 genes, 58.7% of its total up-regulated set), with a comparatively small 8 dpi-unique contribution (228 genes, 5.0%) and minimal carryover from 8 dpi into later stages (38 and 74 genes shared with 16 and 24 dpi, respectively). A core of 521 genes (11.5%) was up-regulated across all three timepoints. FoaER10’s down-regulation followed a similar late/sustained pattern, dominated by genes shared between 16 and 24 dpi (379 genes, 39.0%) and a three-timepoint core of 267 genes (27.5%).

FoaER20 showed the opposite temporal profile: its up-regulated response was overwhelmingly early and largely transient, with 1,697 genes (49.1% of its total up-regulated set) unique to 8 dpi alone, and only 149 genes (4.3%) shared across all three timepoints. In contrast, its down-regulated response was concentrated almost entirely in later infection: no genes were uniquely down-regulated at 8 dpi alone (0 genes, 0.0%), while 645 genes (41.4%) were unique to 24 dpi and a further 712 genes (45.7%) were shared between 16 and 24 dpi, indicating that FoaER20 initiates a large early transcriptional induction but delays essentially all of its repressive response until mid-to-late infection.

FoaZG10 displayed a broad, sustained early-to-mid up-regulation profile, with substantial unique contributions at both 8 dpi (1,411 genes, 28.4%) and 16 dpi (1,582 genes, 31.8%), along with meaningful carryover between these two stages (1,064 genes, 21.4%, shared between 8 and 16 dpi) and a three-timepoint core of 306 genes (6.2%). This response contracted notably by 24 dpi, with only 269 genes (5.4%) uniquely up-regulated at that stage. Its down-regulated response, like the other two isolates, was late-dominant, with the largest single contribution unique to 24 dpi (908 genes, 42.9%).

Overall, contrary to a model of strictly compartmentalized, non-overlapping stage-specific programs, all three isolates maintained a measurable core of genes consistently regulated across their full sampled time course (521, 149, and 306 genes up-regulated in FoaER10, FoaER20, and FoaZG10, respectively; 267, 54, and 230 genes down-regulated). What differed most sharply between isolates was the timing of their dominant transcriptional activity: FoaER10 and FoaZG10 concentrated the bulk of their up-regulated response in later stages (16–24 dpi), whereas FoaER20’s up-regulated response was concentrated early (8 dpi) and its down-regulated response almost exclusively late (16–24 dpi), indicating a temporally front-loaded induction phase followed by a distinct, delayed repression phase.

### Functional-category association of DEGs across curated GO terms

To investigate the molecular functions underlying the transcriptional responses of the three Foa isolates, we examined the representation of DEGs among a curated, of these five categories, only three yielded terms significantly associated with DEGs in this dataset, membrane transport, cell wall degradation/CAZymes, and detoxification/redox, while no significant terms were recovered for cell wall biosynthesis/remodeling or secondary metabolism/toxin biosynthesis. FoaER10 showed seven significantly associated terms, while FoaER20 and FoaZG10 each showed nine (**Figure 5**; **Supplementary File 4**).

**Figure 5:**
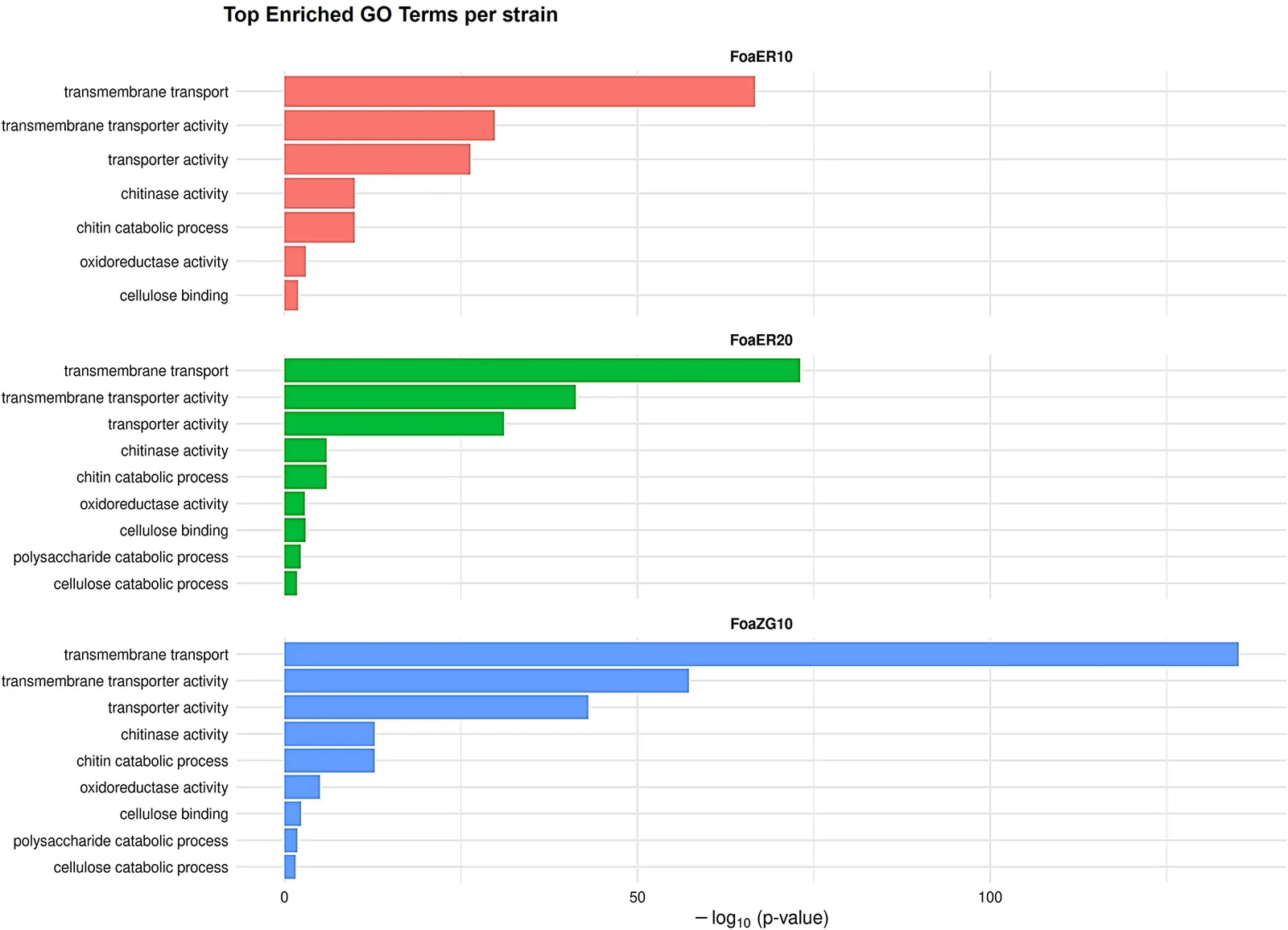
Top enriched Gene Ontology (GO) terms among DEGs across Foa isolates. Enrichment profiles are grouped by isolate: FoaER10 (top, red), FoaER20 (middle, green), and FoaZG10 (bottom, blue). Bar length indicates the statistical significance of enrichment expressed as $-\log_{10}(P\text{-value})$ for each functional category.

Across all three isolates, membrane transport was the most strongly and consistently associated category. Transmembrane transport was associated with 77 genes in FoaER10 (p = 2.09×10^-67^), 81 genes in FoaER20 (p = 8.88×10^-74^), and 147 genes in FoaZG10 (p = 6.63×10^-186^) the single largest gene count and the most significant association observed anywhere in this analysis. Combined across all three transport-related terms (transmembrane transport, transmembrane transporter activity, and transporter activity), FoaZG10 showed the broadest transport response overall (249 genes total), compared with 150 in FoaER20 and 136 in FoaER10. Within this category, up-regulation predominated in nearly every term and isolate, with one notable exception: transmembrane transporter activity in FoaER10 was the only transport-related term in the entire dataset in which down-regulated genes outnumbered up-regulated ones (18 down vs. 12 up) (**Figure 6**).

**Figure 6:**
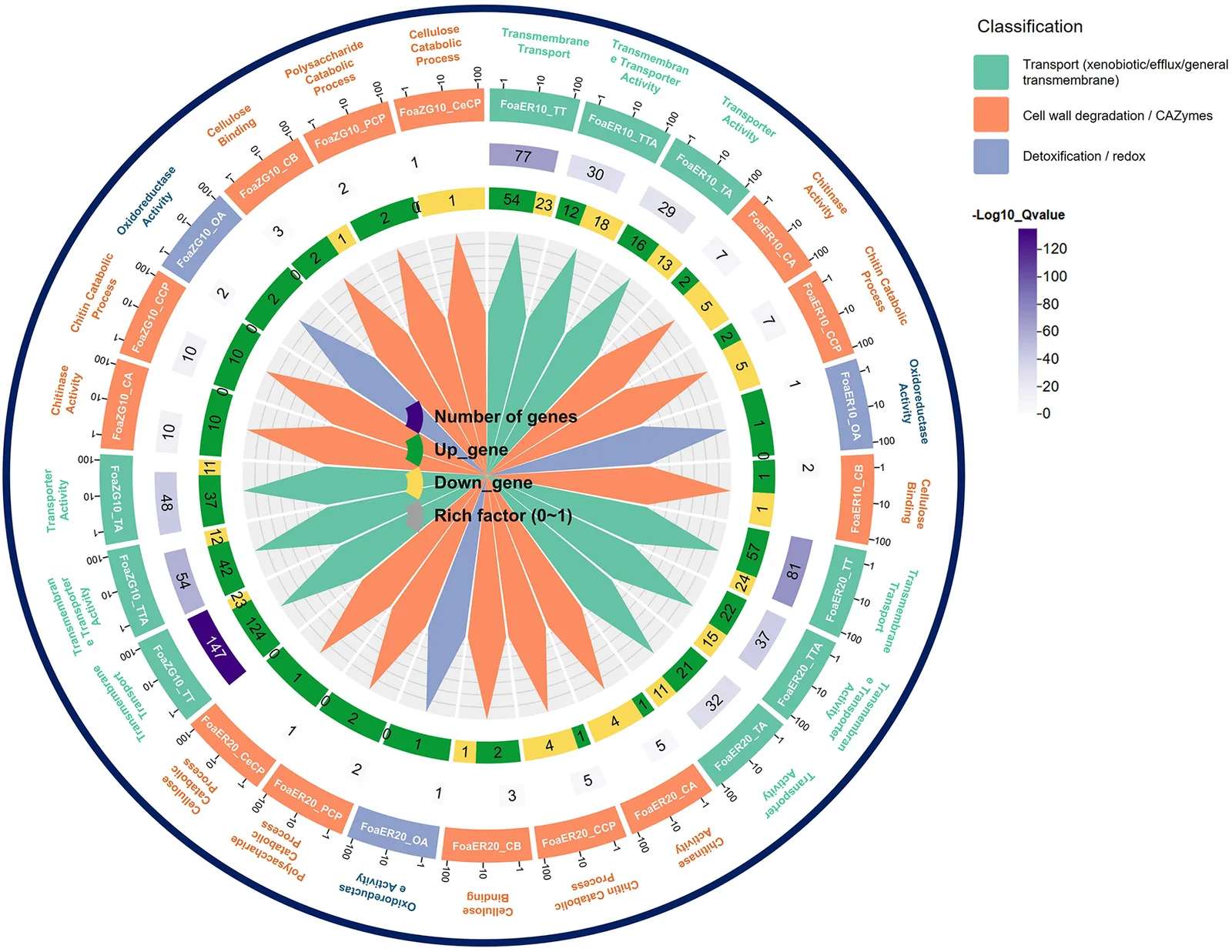
Circos plot summarizing enriched functional categories, DEG counts, and enrichment metrics across Foa isolates. Isolate-specific GO terms are arranged along the perimeter and color-coded by functional classification: Transport (teal), Cell wall degradation/CAZymes (orange), and Detoxification/redox (purple). Moving from the outer perimeter toward the center, the concentric tracks represent: Statistical Significance: Statistical confidence of enrichment ( $-\log_{10}Q\text{-value}$, purple tiles). The total number of differentially expressed genes associated with each term (green/yellow numbered ring). up-regulated genes are shown in orange spikes versus down-regulated in green spikes.

Cell wall degradation/CAZyme terms were also represented in all three isolates, most consistently through chitinase activity and chitin catabolic process, which returned identical gene counts and p-values in every isolate (indicating that these two terms are supported by the same underlying gene set in this dataset, rather than independent evidence). This category showed the clearest isolate-specific reversal in regulation direction: chitinase activity/chitin catabolic process genes were predominantly down-regulated in FoaER10 (2 up, 5 down) and FoaER20 (1 up, 4 down), but were exclusively up-regulated in FoaZG10 (10 up, 0 down), directly consistent with the isolate-specific direction reversal already described for individual chitinase genes (e.g., Endochitinase B1) elsewhere in this study. Cellulose binding was significant in all three isolates (1–2 genes each), while polysaccharide catabolic process and cellulose catabolic process reached significance only in FoaER20 and FoaZG10, not in FoaER10.

Detoxification/redox representation, captured exclusively by oxidoreductase activity, was present in all three isolates but consistently contributed the smallest gene counts of any significant category (1 gene in FoaER10 and FoaER20, 2 genes in FoaZG10), and was entirely up-regulated in every isolate.

Overall, the three isolates showed a broadly shared set of enriched functional terms rather than isolate-specific functional specialization, differing primarily in the magnitude of association (particularly for membrane transport, where FoaZG10 showed a markedly larger response) and, notably, in the direction of regulation within the cell wall degradation/CAZyme category, where FoaER10 and FoaER20 showed predominantly repressed chitinase-related genes while FoaZG10 showed the same functional category exclusively induced. These values describe the number and functional identity of DEGs associated with each term and are reported descriptively; they should not be read as evidence of statistical over-representation relative to genome background beyond the significance threshold already applied in the underlying enrichment analysis.

### Validation of RNA-seq gene expression profiles by RT-qPCR

To validate the RNA-seq results and confirm the expression of biologically relevant candidates, four genes were selected for RT-qPCR confirmation based on two complementary criteria (**Figure 7**). SIX1 (H9L39_19845) was selected as it ranked among the most consistently and highly expressed genes shared across all three isolates in the transcriptomic dataset, identifying it as part of the conserved pathogenicity core (Supp. File 5). It belongs to the SIX gene family, which encodes small cysteine-rich effector proteins that are translocated into the host vascular system and are well-characterized virulence determinants in the *F. oxysporum*-tomato pathosystem. In contrast, H9L39_17240 (SIX9), H9L39_15275 (NLP), and H9L39_04485 (NLP) were selected because they exhibited pronounced isolate-specific divergence in expression across the RNA-seq dataset: SIX9 reversed direction entirely between isolates (strongly down-regulated in FoaER10, strongly up-regulated in FoaER20 and FoaZG10); H9L39_15275 was up-regulated in FoaER20 but down-regulated in the other two isolates; and H9L39_04485 was up-regulated in all three isolates, but to markedly different degrees. H9L39_15275 and H9L39_04485 are orthologs of necrosis and ethylene-inducing peptide 1-like proteins (NLPs), a class of cytotoxic effectors that disrupt host membrane integrity and trigger cell death responses in a broad range of plant-pathogen interactions.

**Figure 7:**
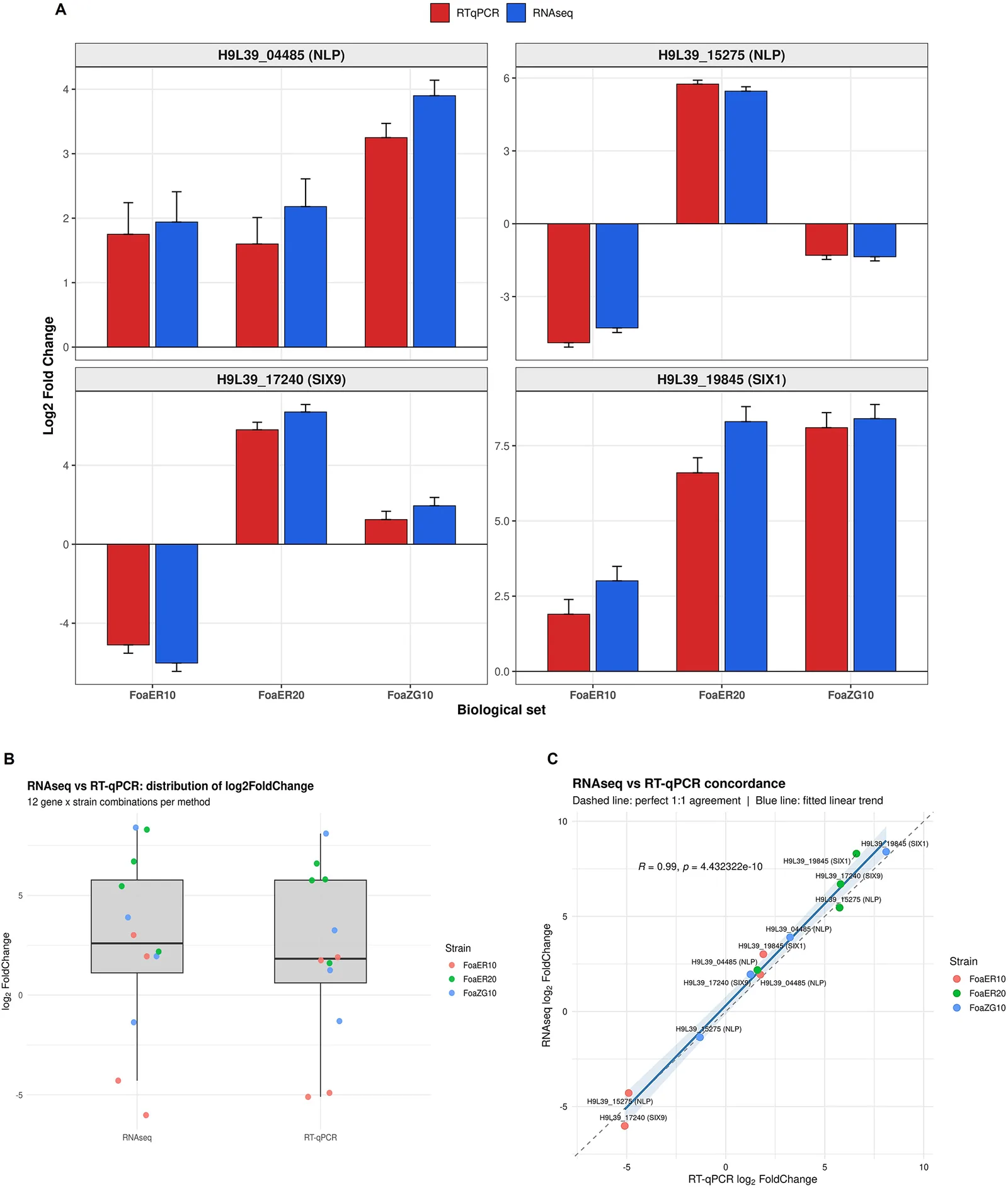
Validation and concordance of RNA-seq expression profiles with RT-qPCR. **(A)** Bar plots comparing the ${\text{log}}_{2}$ Fold Change ( $\log_{2}\text{FC}$) values for four candidate effector genes, two NLP homologues (H9L39_04485 and H9L39_15275) and two SIX homologues (H9L39_17240/SIX9 and H9L39_19845/SIX1), across three Foa isolates (FoaER10, FoaER20, and FoaZG10). Red bars depict targeted quantification by RT-qPCR (normalized against the ACTIN reference gene), while blue bars depict RNA-seq $\log_{2}\text{FC}$ values. Error bars indicate standard error/deviation across biological replicates. **(B)** Box plots with overlaid individual data points displaying the distribution of $\log_{2}\text{FC}$ values across 12 gene × strain combinations for both RNA-seq and RT-qPCR, color-coded by strain. **(C)** Correlation scatter plot demonstrating strong expression concordance between RT-qPCR (*x*-axis) and RNA-seq (*y*-axis) across all evaluated conditions ( R=0.99, $p=4.43\times 10^{-10}$). The dashed diagonal line denotes perfect 1:1 agreement, and the solid blue line represents the fitted linear regression model with a 95% confidence interval.

All isolates exhibited consistent up-regulation of SIX1, with FoaER20 (6.6 ± 0.5) and FoaZG10 (8.1 ± 0.5) exhibiting higher expression levels than FoaER10 (1.9 ± 0.49). This pattern indicates that SIX1 may have a conserved function during host colonization. SIX1 is homologous to the Avr3 effector found in the Fol–tomato pathosystem, where it is linked to host pathogen recognition; however, its exact role in the date palm pathosystem still needs to be tested. SIX9, by contrast, showed the most dramatic isolate-specific divergence of the four validated genes, being strongly down-regulated in FoaER10 (-5.1 ± 0.42) while strongly up-regulated in FoaER20 (5.8 ± 0.38) and moderately up-regulated in FoaZG10 (1.25 ± 0.42), a complete reversal in regulation direction rather than simply “lower or more variable” expression, indicating that SIX9 is subject to strong isolate-specific regulation rather than belonging to a conserved response. H9L39_15275 was strongly up-regulated in FoaER20 (5.75 ± 0.16) but down-regulated in both FoaER10 (-4.9 ± 0.18) and FoaZG10 (-1.3 ± 0.17), suggesting that this necrosis-inducing effector may contribute specifically to the virulence strategy of FoaER20. Conversely, H9L39_04485 showed moderate up-regulation in all three isolates (1.75 ± 0.49, 1.60 ± 0.41, and 3.25 ± 0.22 in FoaER10, FoaER20, and FoaZG10, respectively). Together, the divergent regulation of SIX9 and H9L39_15275 across isolates suggests a potential contribution of these genes, and others with similar divergent expression patterns, to the distinct infection strategies observed among Foa isolates as shown in individual gene expression comparisons (**Figure 7A**) and log2 fold-change distributions (**Figure 7B**).

Across all four genes and three isolates (12 gene × strain measurements), RT-qPCR and RNA-seq log2 fold-change estimates showed strong concordance (Pearson r = 0.99, p = 4.4×10^-^¹^0^; **Figure 7C**), confirming that the RNA-seq-derived expression patterns discussed throughout this study are independently reproducible by an orthogonal method.

## Discussion

Successful host colonization by fungal pathogens requires coordinated regulation of genes involved in metabolism, host penetration, and interaction with plant defense mechanisms. In this study, we performed a comparative temporal transcriptomic analysis of three Foa isolates during infection of date palm roots to investigate the molecular basis of Foa pathogenicity. Previous genomic studies have predicted several pathogenicity-associated components in Foa and demonstrated the presence of fungal transcripts in infected tissues (Khayi et al., 2023; Mbaye et al., 2026; Rafiqi et al., 2022). However, transcriptomic analyses capturing temporal gene expression dynamics during infection have not been reported for this pathosystem. In contrast, similar approaches in other *F. oxysporum* pathosystems, including melon, *Medicago truncatula*, and cucumber, have revealed extensive transcriptional reprogramming associated with infection processes (Huang et al., 2019; Thatcher et al., 2016; Yang et al., 2022).

Analysis of gene expression across the three infection stages (8, 16, and 24 dpi, relative to the 0 dpi baseline) revealed extensive but isolate-specific transcriptional dynamics rather than a single, shared temporal trajectory. FoaER10 showed a progressive, cumulative increase in the number of differentially expressed genes across the time course (1,250 DEGs at 8 dpi, rising to 4,313 at 16 dpi and 4,723 at 24 dpi), consistent with a gradually intensifying transcriptional response as infection progressed. FoaER20, by contrast, showed its largest response at 8 dpi (2,726 DEGs) followed by a progressive contraction at 16 dpi (2,438 DEGs) and 24 dpi (1,832 DEGs), indicating an early, front-loaded transcriptional induction rather than a delayed or mid-infection peak. FoaZG10 showed a broad, sustained response from 8 dpi (3,549 DEGs) through its own peak at 16 dpi (4,028 DEGs), followed by a partial contraction at 24 dpi (2,575 DEGs). Similar isolate-to-isolate variability the timing of transcriptional activity has been reported among *Fusarium oxysporum* f. sp. *cucumerinum* isolates has been reported in other studies and may reflect differences in adaptation to host environments or infection strategies (Huang et al., 2019). As these temporal profiles were generated through independent DESeq2 models fitted separately for each isolate, comparing the absolute number of DEGs across isolates provides exploratory insight into differing temporal strategies rather than formal statistical evidence of an isolate-by-time interaction; because baseline expression and dispersion can vary across isolates modeled independently, future studies employing integrated factorial designs (isolate × time interaction models) would be valuable to formally test for genotype-by-time interaction effects during host colonization.

Functional annotation of pathogenicity-associated DEGs revealed a set of enriched functional categories that was, notably, largely shared across the three isolates rather than sharply isolate-specific. Gene Ontology enrichment analysis, restricted to genes already flagged as pathogenicity-associated (priority CAZymes, MFS transporters, NLP/Hce2 effector homologs, lipase_3 and abhydrolase domains) and significantly differentially expressed in each isolate, identified consistent enrichment for membrane transport (transmembrane transport, transmembrane transporter activity, transporter activity), cell wall-degrading CAZyme activity (chitinase activity, chitin catabolic process, cellulose binding), and oxidoreductase/detoxification activity in all three isolates. FoaER20 and FoaZG10 additionally showed enrichment for polysaccharide catabolic process and cellulose catabolic process, which were not significantly enriched in FoaER10. No isolate showed significant enrichment for cell wall biosynthesis/remodeling or secondary metabolism/toxin-related terms among its pathogenicity-associated DEGs in this analysis; the shared enrichment for transport and CAZyme-mediated cell wall degradation across all three isolates, rather than pointing to categorically distinct isolate-specific strategies, suggests that these processes represent a conserved core of the Foa transcriptional response to host colonization, consistent with roles described for cell wall-degrading enzymes and membrane transporters in facilitating host penetration, colonization, and metabolite exchange in other plant-pathogenic fungi (R. King et al., 2017; Liu and Liu, 2016; Scott-Craig et al., 1998). Major facilitator superfamily (MFS) transporters were the single largest category of pathogenicity-associated genes identified across the dataset as a whole, and were also the most frequently represented category among the individual pathogenicity genes highlighted in the volcano plot analysis (**Figure 3**), underscoring the prominence of membrane transport in this response.

The identification of candidate pathogenicity-associated effector genes, including SIX proteins and NLPs, provides further insight into host-pathogen transcriptional dynamics. SIX1 (H9L39_19845) and SIX9 (H9L39_17240) showed differential expression across all three isolates, consistent with a potential involvement in colonization, as reported for homologs in other Fusarium species (Houterman et al., 2007; Rocafort et al., 2020). Rather than a simple progressive increase, SIX1 showed a consistent peak in expression at 16 dpi across all three isolates (log2FC = 3.01, 8.30, and 8.49 in FoaER10, FoaER20, and FoaZG10, respectively), with expression declining or reversing to down-regulation by 24 dpi in FoaER10 and FoaER20 (log2FC = −7.82 and −3.90) while remaining up-regulated in FoaZG10 (log2FC = 2.75). This pattern was independently confirmed by RT-qPCR (**Figure 7**; Pearson r = 0.99 between RNA-seq and RT-qPCR log2 fold-change estimates across the four validated genes). SIX9 showed a more isolate-divergent pattern, remaining consistently down-regulated across all three time points in FoaER10 (log2FC = −9.30, −6.02, and −6.02 at 8, 16, and 24 dpi) while showing an early-to-mid induction in FoaER20 and FoaZG10. Two NLP genes were similarly examined: H9L39_15275 was up-regulated wherever significantly detected in all three isolates (log2FC ranging from 1.46 to 6.56), whereas H9L39_04485 showed isolate-specific divergence in regulation direction, being significantly down-regulated in FoaER10 (log2FC = −4.29 at 16 dpi) but significantly up-regulated in FoaER20 (log2FC = 5.46 at 16 dpi) and, depending on time point, in FoaZG10 (up at 8 dpi, log2FC = 5.23, down at 16 dpi, log2FC = −1.36). This isolate-dependent reversal in regulation direction for H9L39_04485 is consistent with the broader pattern identified through Venn diagram analysis, in which 2,376 genes were found to be up-regulated in at least one isolate but down-regulated in another. This differential, and in some cases opposing, expression within the NLP gene family may reflect divergence in transcriptional regulation, as previously reported in other fungal pathogens (Pour et al., 2020). Although NLPs are known to induce necrosis and contribute to disease development in other systems (Bailey, 1995), their specific roles in the Foa-date palm interaction remain to be experimentally validated. While the differential expression of candidate genes such as SIX and NLP homologs points to their potential involvement in host colonization, transcript abundance alone does not establish a definitive effector function or virulence mechanism; future functional characterization, such as targeted gene disruption and quantitative disease assays, will be required to confirm their biological roles during infection.

Because this study is transcriptomic rather than functional, the roles proposed for these candidate effectors rest on homology to previously characterized proteins rather than direct experimental confirmation in Foa. SIX1 (H9L39_19845) is homologous to Avr3 of F. oxysporum f. sp. lycopersici, which is secreted into xylem sap and recognized by the tomato resistance protein I-3 (Rep et al., 2004), and a SIX1 homolog in F. oxysporum f. sp. cubense TR4 required for full virulence on banana, as show by targeted gene deletion (Widinugraheni et al., 2018). The strong, conserved up-regulation of SIX1 across all three Foa isolates, peaking during mid-to-late colonisation, is consistent with a similar virulence-associated role, although whether Foa SIX1 is required for virulence or recognized by an as-yet-uncharacterized date palm resistance protein remains untested. SIX9 (H9L39_17240) is less functionally characterized individually, but SIX-family gene as a class are consistently among the most infection-induced transcripts in xylem-colonizing Fusarium formae speciales, including F. oxysporum f. sp. pisi (Jenkins et al., 2021); then near-complete reversal in its regulation direction between FoaER10 and the other two isolates observed here has no clear precedent in the published literature, to the best of our knowledge, and my reflect isolate-specific rewiring of effector deployment. The two candidate NLP effectors (H9L39_15275, H9L39_04485) belong to a family first shown to induce necrosis and ethylene production when purified directly from F. oxysporum culture filtrate (Bailey, 1995); Structural work has since shown that this necrotizing activity derives from a conserved toxin fold that permeabilizes host membranes by binding plant-specific sphingolipids (Ottmann et al., 2009), offering a concrete mechanistic basis for their potential contribution to the vascular tissue damage characteristic of Foa infection. The opposing regulation of H9L39_04485 across isolates parallels the isolate-dependent divergence noted for SIX9, suggesting coordinated, isolate-specific control of necrotizing and colonization-associated effectors, though this remains speculative without paired cell-death assays on infected date palm tissue. The Hce2-family effector identified here (H9L39_03256) is homologous to Ecp1/Ecp2 of *Cladosporium fulvum*, which are required for full virulence tomato (Laugé et al., 1997), and belongs to a broader Hce2 superfamily conserved across fungi infecting both dicot and monocot hosts. Some distantly related homologs can also be recognized by the same tomato Cf-Ecp2 resistance protein despite this taxonomic distance (Stergiopoulos et al., 2010, Stergiopoulos et al., 2012). This makes it a priority candidate for for future recognition and virulence studies in date palm. Taken together, homology to functionally validated effectors in order pathosystems provides plausible mechanic context for the candidates identified here, but transcript abondance alone does not establish effector function or host recognition in Foa; targeted gene disruption, complementation, and infection or necrosis assays will be needed to confirm these roles.In this study, bioinformatic sequence filtering was optimized specifically to maximize fungal transcript recovery and elucidate pathogen-centric virulence dynamics. Although host transcriptomics were outside the scope this pathogen-targeted sequencing design, these findings provide insight into the fungal side of the host-pathogen interface. It should be noted that the host material comprised seedlings of cv. Boufgouss derived from open-pollinated seed, a genetically heterogeneous background; rather than pooling root tissue across seedlings to average out this heterogeneity, each seedling was treated as an independent biological replicate and sampled and sequenced individually (n = 3 seedlings per isolate-time point combination, no pooling), so that this genetic variability is captured within, rather than masked from, the observed biological replicate variance. Future functional assays and effector characterizations would nonetheless benefit from genetically uniform, micropropagated clonal date palm material to reduce this source of variance further. The temporal activation of Foa candidate effectors and cell wall-degrading enzymes observed here coincides with known *Phoenix dactylifera* defense responses documented in the literature; date palm infection by Foa has been reported to trigger host defense mechanisms including activation of the phenylpropanoid pathway, accumulation of pathogenesis-related (PR) proteins, and vascular lignification, and the induction of fungal effectors and CAZymes at 16–24 dpi observed here may reflect an active counter-defense response to these host immune pathways. Future investigations incorporating dedicated dual RNA-seq designs, sampling host and pathogen transcriptomes simultaneously from the same infected tissue, will be needed to directly resolve this host-pathogen co-transcriptional dynamic.

Taken together, the functional enrichment analysis performed here, restricted to Gene Ontology terms among pathogenicity-associated genes, indicates that membrane transport, CAZyme-mediated cell wall degradation, and oxidoreductase/detoxification activity constitute a conserved core of the Foa transcriptional response to host colonization across all three isolates studied. KEGG pathway enrichment was not performed in this study, as it requires Kegg Orthology (KO) assignment via a dedicated annotation step (eggNOG-mapper) that has not yet been carried out for this genome assembly; a more complete picture of the metabolic pathways engaged during Foa colonization, including possible contributions from primary metabolism, protein secretion, and other cellular processes beyond the pathogenicity-associated gene set examined here, will require this additional annotation and a corresponding genome-wide enrichment analysis. These findings should accordingly be interpreted as an initial, targeted characterization of the pathogenicity-associated transcriptional response rather than a comprehensive account of Foa’s full metabolic reprogramming during infection, and align with established general models of fungal pathogenesis involving coordinated deployment of cell wall-degrading enzymes and membrane transporters during host colonization (Roncero et al., 2003; Williams et al., 2016).

While this study provides a comprehensive transcriptomic map of Foa gene expression during date palm colonization, the functional roles of the candidate genes identified remain to be experimentally validated. An additional limitation is that, for each isolate, all biological replicates across all four time points were inoculated from a single, shared conidial suspension rather than from independently prepared inoculum batches; while this ensures consistent inoculum concentration and composition across replicates and time points within an isolate, it means that inoculum-batch variation is not captured within the observed replicate-to-replicate variance, and true inoculum-level biological replication was not achieved within a given isolate. Transcriptomic profiling reveals correlative patterns of gene regulation but cannot establish causality between gene expression and virulence phenotypes. Future work should therefore prioritize targeted functional characterization of the most promising candidates including the conserved core pathogenicity genes, isolate-specific effectors such as necrosis-inducing proteins and SIX genes, and stage-specific regulators highlighted in this study through approaches such as gene deletion, overexpression, or heterologous complementation, combined with pathogenicity bioassays in date palm. Integrating such functional data with phenotypic disease progression measurements and host transcriptomic responses will be essential to fully elucidate the molecular basis of Bayoud disease.

## Conclusion

This study provides the first comprehensive *in planta* transcriptomic characterization of Foa during date palm root colonization, revealing dynamic and stage-dependent transcriptional reprogramming across three isolates. The integration of differential expression analysis, heatmap clustering, and functional annotation highlights extensive modulation of genes associated with membrane transport, cell wall degradation, and oxidoreductase/detoxification activity, particularly among pathogenicity-associated genes at mid- and late-infection stages. Several candidate pathogenicity genes were identified, including priority CAZymes, major facilitator superfamily (MFS) transporters, necrosis-inducing-like proteins (NLPs), a Hce2/Ecp2-family effector, and SIX effectors (SIX1 and SIX9); several of these, including SIX1 and one NLP homolog, showed isolate-dependent reversal in regulation direction across the infection time course, in addition to differences in timing and magnitude. Comparative analysis across isolates further identified a conserved core of genes consistently regulated across all three isolates, alongside substantial isolate-specific and, in some cases, direction-reversed responses, indicating that Foa isolates employ both shared and divergent transcriptional strategies during host colonization. Overall, this study provides a valuable transcriptomic resource for understanding the molecular basis of the Foa-date palm interaction and identifies a set of promising candidate genes for future investigation. Functional validation approaches will be essential to establish the roles of these genes in pathogenicity and to support the development of effective and sustainable management strategies for Bayoud disease.

## Data Availability

The RNA sequencing datasets generated in this study have been deposited in the NCBI Sequence Read Archive (SRA) under the BioProject accession numbers PRJNA1422174 (FoaER10), PRJNA1491631 (FoaER20), and PRJNA1422436 (FoaZG10).

## References

[B1] Ayada H. Dhioui B. Mazouz H. El harrak A. Jaiti F. Ouhmidou B. . (2022). In silico comparative genomic analysis unravels a new candidate protein arsenal specifically associated with Fusarium oxysporum f. sp. albedinis pathogenesis. Sci. Rep. 12, 1–18. doi: 10.1038/s41598-022-21858-1 36351932 PMC9646873

[B2] Baayen R. P. O’Donnell K. Bonants P. J. M. Cigelnik E. Kroon L. P. N. M. Roebroeck E. J. A. . (2000). Gene genealogies and AFLP analyses in the Fusarium oxysporum complex identify monophyletic and nonmonophyletic formae speciales causing wilt and rot disease. Phytopathology 90, 891–900. doi: 10.1094/PHYTO.2000.90.8.891 18944511

[B3] Bailey B. A. (1995). Purification of a protein from culture filtrates of Fusarium oxysporum that induces ethylene and necrosis in leaves of Erythroxylum coca. Phytopathology 85, 1250–1255. doi: 10.1094/PHYTO-85-1250

[B4] Benzohra I. E. Megateli M. Berdja R. (2015). Bayoud disease of date palm in Algeria: History, epidemiology and integrated disease management. Afr. J. Biotechnol. 14 (7), 542–550. doi: 10.5897/AJBX2014.14292

[B5] Del Sorbo G. Schoonbeek H. J. De Waard M. A. (2000). Fungal transporters involved in efflux of natural toxic compounds and fungicides. Fungal Genet. Biol. 30, 1–15. doi: 10.1006/fgbi.2000.1206 10955904

[B6] De Wit P. J. G. M. Mehrabi R. Van Den Burg H. A. Stergiopoulos I. (2009). Fungal effector proteins: past, present and future. Mol. Plant Pathol. 10, 735–747. doi: 10.1111/J.1364-3703.2009.00591.X 19849781 PMC6640362

[B7] Diouf A. M. Mbaye A. L. Deh M. Barakate M. Rchiad Z. (2025). Whole genome analysis of antagonistic and plant growth promoting Bacillus halotolerans KFD uncovers its molecular arsenal against the bayoud pathogen Fusarium oxysporum f.sp albedinis. Biol. Control 206, 105790. doi: 10.1016/j.biocontrol.2025.10579042574925

[B8] Ferniah R. S. Pujiyanto S. Kusumaningrum H. P. (2014). Characterization and pathogenicity of Fusarium oxysporum as the causal agent of Fusarium wilt in chili (*Capsicum annuum* L.). Microbiol. Indones. 8 (3), 121–126.

[B9] Houterman P. M. Speijer D. Dekker H. L. De Koster C. G. Cornelissen B. J. C. Rep M. (2007). The mixed xylem sap proteome of Fusarium oxysporum-infected tomato plants. Mol. Plant Pathol. 8, 215–221. doi: 10.1111/J.1364-3703.2007.00384.X 20507493

[B10] Huang X. Sun M. Lu X. Li S. (2019). Serial passage through resistant and susceptible cucumber cultivars affects the virulence of Fusarium oxysporum f. sp. cucumerinum. Microbiol. Open 8, e00641. doi: 10.1002/mbo3.641 29797483 PMC6391263

[B11] Jenkins S. Taylor A. Jackson A. C. Armitage A. D. Bates H. J. Mead A. . (2021). Identification and expression of secreted in xylem pathogenicity genes in Fusarium oxysporum f. sp. pisi. Front. Microbiol. 12. doi: 10.3389/FMICB.2021.593140 33897626 PMC8062729

[B12] Jones D. A. Bertazzoni S. Turo C. J. Syme R. A. Hane J. K. (2018). Bioinformatic prediction of plant–pathogenicity effector proteins of fungi. Curr. Opin. Microbiol. 46, 43–49. doi: 10.1016/J.MIB.2018.01.017 29462764

[B13] Jones J. D. G. Dangl J. L. (2006). The plant immune system. Nature 444, 323–329. doi: 10.1038/NATURE05286 17108957

[B14] Khayi S. Armitage A. D. Gaboun F. Meftah-kadmiri I. Lahlali R. Fokar M. . (2023). Chromosome-scale assembly uncovers genomic compartmentation of Fusarium oxysporum f. sp. albedinis, the causal agent of Bayoud disease in date palm. Front. Microbiol. 14, 1268051. doi: 10.3389/FMICB.2023.1268051 37886058 PMC10599148

[B15] King R. Urban M. Lauder R. P. Hawkins N. Evans M. Plummer A. . (2017). A conserved fungal glycosyltransferase facilitates pathogenesis of plants by enabling hyphal growth on solid surfaces. PloS Pathog. 13, e1006672. doi: 10.1371/JOURNAL.PPAT.1006672 29020037 PMC5653360

[B16] King B. C. Waxman K. D. Nenni N. V. Walker L. P. Bergstrom G. C. Gibson D. M. (2011). Arsenal of plant cell wall degrading enzymes reflects host preference among plant pathogenic fungi. Biotechnol. Biofuels 4, 4. doi: 10.1186/1754-6834-4-4 21324176 PMC3051899

[B17] Laugé R. Joosten M. H. A. J. Van Den Ackerveken G. F. J. M. Van Den Broek H. W. J. De Wit P. J. G. M. (1997). The in planta-produced extracellular proteins ECP1 and ECP2 of Cladosporium fulvum are virulence factors. Https://Doi.Org/10.1094/MPMI.1997.10.6.725 10, 725–734. doi: 10.1094/MPMI.1997.10.6.725

[B18] Liu X. Liu C. (2016). Effects of drought-stress on Fusarium crown rot development in Barley. PloS One 11, 1–13. doi: 10.1371/journal.pone.0167304 27936004 PMC5147875

[B19] Livak K. J. Schmittgen T. D. (2001). Analysis of relative gene expression data using real-time quantitative PCR and the 2-ΔΔCT method. Methods 25, 402–408. doi: 10.1006/meth.2001.1262 11846609

[B20] Love M. I. Huber W. Anders S. (2014). Moderated estimation of fold change and dispersion for RNA-seq data with DESeq2. Genome Biol. 15, 550. doi: 10.1186/s13059-014-0550-8 PMC4302049 25516281

[B21] Mbaye A. L. Diouf A. M. Deh M. Ghait H. Khayi S. Lahlali R. . (2026). Comparative genomics of Fusarium oxysporum f. sp. albedinis reveals the effector repertoire and molecular arsenal for date palm infection. BMC Plant Biol. 2026 26:1 26, 940. doi: 10.1186/S12870-026-08659-8 42010430 PMC13224452

[B22] Oome S. Van Den Ackerveken G. (2014). Comparative and functional analysis of the widely occurring family of Nep1-like proteins. Mol. Plant-Microbe Interactions : MPMI 27, 1081–1094. doi: 10.1094/MPMI-04-14-0118-R 25025781

[B23] Ottmann C. Luberacki B. Küfner I. Koch W. Brunner F. Weyand M. . (2009). A common toxin fold mediates microbial attack and plant defense. Proc. Natl. Acad. Sci. 106, 10359–10364. doi: 10.1073/PNAS.0902362106 19520828 PMC2695407

[B24] Pour F. N. Cobos R. Coque J. J. R. Serôdio J. Alves A. Félix C. . (2020). Toxicity of recombinant necrosis and ethylene-inducing proteins (NLPs) from Neofusicoccum parvum. Toxins 2020 Vol. 12 Page 235 12, 235. doi: 10.3390/TOXINS12040235 32272814 PMC7232490

[B25] Rafiqi M. Jelonek L. Diouf A. M. Mbaye A. L. Rep M. Diarra A. (2022). Profile of the in silico secretome of the palm dieback pathogen, Fusarium oxysporum f. sp. albedinis, a fungus that puts natural oases at risk. PloS One 17, 1–14. doi: 10.1371/journal.pone.0260830 35617325 PMC9135196

[B26] Rep M. Van Der Does H. C. Meijer M. Van Wijk R. Houterman P. M. Dekker H. L. . (2004). A small, cysteine-rich protein secreted by Fusarium oxysporum during colonization of xylem vessels is required for I-3-mediated resistance in tomato. Mol. Microbiol. 53, 1373–1383. doi: 10.1111/j.1365-2958.2004.04177.x 15387816

[B27] Rocafort M. Fudal I. Mesarich C. H. (2020). Apoplastic effector proteins of plant-associated fungi and oomycetes. Curr. Opin. Plant Biol. 56, 9–19. doi: 10.1016/j.pbi.2020.02.004 32247857

[B28] Roncero M. I. G. Hera C. Ruiz-Rubio M. García Maceira F. I. Madrid M. P. Caracuel Z. . (2003). Fusarium as a model for studying virulence in soilborne plant pathogens. Physiol. Mol. Plant Pathol. 62, 87–98. doi: 10.1016/S0885-5765(03)00043-2

[B29] Stergiopoulos I. Kourmpetis Y. A. I. Slot J. C. Bakker F. T. De Wit P. J. G. M. Rokas A. (2012). In silico characterization and molecular evolutionary analysis of a novel superfamily of fungal effector proteins. Mol. Biol. Evol. 29, 3371–3384. doi: 10.1093/MOLBEV/MSS143 22628532

[B30] Stergiopoulos I. Van Den Burg H. A. Ökmen B. Beenen H. G. Van Liere S. Kema G. H. J. . (2010). Tomato Cf resistance proteins mediate recognition of cognate homologous effectors from fungi pathogenic on dicots and monocots. PNAS 107, 7610–7615. doi: 10.1073/pnas.1002910107 20368413 PMC2867746

[B31] Tantaoui A. Ouinten M. Geiger J. P. Fernandez D. (1996). Characterization of Fusarium oxysporum f. sp. albedinis isolated from date palms using restriction fragment length polymorphisms and RAPD markers. Phytopathology 86, 781–788. doi: 10.1094/Phyto-86-781

[B32] Thatcher L. F. Williams A. H. Garg G. Buck S. A. G. Singh K. B. (2016). Transcriptome analysis of the fungal pathogen Fusarium oxysporum f. sp. medicaginis during colonisation of resistant and susceptible Medicago truncatula hosts identifies differential pathogenicity profiles and novel candidate effectors. BMC Genomics 17 (1), 860. doi: 10.1186/S12864-016-3192-2 27809762 PMC5094085

[B33] Widinugraheni S. Niño-Sánchez J. Van Der Does H. C. Van Dam P. García-Bastidas F. A. Subandiyah S. . (2018). A SIX1 homolog in Fusarium oxysporum f.sp. cubense tropical race 4 contributes to virulence towards Cavendish banana. PloS One 13, e0205896. doi: 10.1371/JOURNAL.PONE.0205896 30346962 PMC6197647

[B34] Williams A. H. Sharma M. Thatcher L. F. Azam S. Hane J. K. Sperschneider J. . (2016). Comparative genomics and prediction of conditionally dispensable sequences in legume-infecting Fusarium oxysporum formae speciales facilitates identification of candidate effectors. BMC Genomics 17 (1), 191. doi: 10.1186/S12864-016-2486-8 26945779 PMC4779268

[B35] Yang T. Liu J. Li X. Amanullah S. Lu X. Zhang M. . (2022). Transcriptomic analysis of Fusarium oxysporum stress-induced pathosystem and screening of Fom-2 interaction factors in contrasted melon plants. Front. Plant Sci. 13, 961586. doi: 10.3389/FPLS.2022.961586 35937314 PMC9354789

[B36] Young M. D. Wakefield M. J. Smyth G. K. Oshlack A. (2010). Gene ontology analysis for RNA-seq: accounting for selection bias. Genome Biol. 11, 1–12. doi: 10.1186/GB-2010-11-2-R14 20132535 PMC2872874

