# Supplementary material for: Transcriptomic analysis of the fungal pathogen Fusarium oxysporum f. sp. albedinis (Foa) during colonization of date palm reveals distinct temporal transcriptional trajectories and uncovers novel candidate effectors
Source: Front Plant Sci. 2026 Oct 21;17:1954982. doi: 10.3389/fpls.2026.1954982 (PMC13731343; doi:10.3389/fpls.2026.1954982)
Supplement: Supplementary file 1 [file DataSheet2.docx]

| Isolate ID | FOA28/TL3 Diagnostic PCR | NCBI BioProject Accession | Host Virulence (P. dactylifera) | Phenotypic Aggressiveness / DSI Rank |
| --- | --- | --- | --- | --- |
| FoaER10 | Confirmed Positive (100% ID) | SAMN49516559 | Virulent | Rank 1 (Highest DSI, shortest incubation) |
| FoaER20 | Confirmed Positive (100% ID) | SAMN49517112 | Virulent | Rank 2 (High DSI) |
| FoaZG10 | Confirmed Positive (100% ID) | SAMN49517222 | Virulent | Rank 3 (Moderate DSI) |

Table 1. Molecular authentication, genomic accessions, and comparative pathogenicity metrics of *Fusarium oxysporum* f. sp. *albedinis* isolates on date palm (*Phoenix dactylifera*) seedlings.

Figure 1. Distribution, correlation, and group-wise comparison of sequencing quality metrics. (A) Violin plots showing the distribution of core quality metrics (Effective %, Q20 %, Q30 %, and GC %) across the three experimental groups (FoaER10, FoaZG10, and FoaER20), with embedded box plots marking the median and interquartile range. (B) Pearson correlation matrix of the quality metrics, with red indicating positive and blue indicating negative correlation. Q20 and Q30 scores correlate strongly with GC content, and both correlate inversely with error rate, as expected. (C) Mean values of each quality metric by treatment group, showing consistently high sequencing quality across all conditions (Q30 ≥ 95% throughout). (D) Key sequencing parameters tracked across infection stages (0, 8, 16, and 24 d.p.i.). From left to right: effective rate (%), reflecting the proportion of usable, high-quality data; read loss (%), reflecting filtering stringency; and error rate (%), reflecting sequencing accuracy. Error bars indicate SEM across replicates.
